# The Cross-Talk Between Atopic Dermatitis and Diabetes Mellitus: A Meta-Analysis

**DOI:** 10.7759/cureus.13750

**Published:** 2021-03-07

**Authors:** Hyder O Mirghani, Khalid Alhazmi, Salah Alghamdi, Mohammed Alraddadi

**Affiliations:** 1 Internal Medicine, University of Tabuk, Tabuk, SAU; 2 Pathology, University of Tabuk, Tabuk, SAU; 3 Surgery, University of Tabuk, Tabuk, SAU

**Keywords:** atopic dermatitis, diabetes mellitus, prevalence.

## Abstract

Introduction: Atopic dermatitis (AD) is associated with various systemic diseases. However, its association with diabetes mellitus (DM) was discussed controversially. Few researchers reviewed the association of these two common morbid disorders. This meta-analysis aimed to assess the relationship between AD and DM.

Methods: We systematically searched PubMed including Epub and ahead of print (198 articles identified) and Cochrane (13 articles) databases. The searching engine was set to include case-control, prospective and retrospective cohorts, and cross-sectional studies from the first published up to February 12, 2021. Two hundred and eleven were identified, eighteen full texts were screened; of them, six were included in the final meta-analysis. The keywords used were AD, diabetes mellitus, type 1 diabetes, and type 2 diabetes. A datasheet was used to record the author's name, year of publication, country and type of the studies, number of events, and total number in the two arms (patients and controls).

Results: Out of the 211 references identified, six studies were pooled to test the association between diabetes mellitus and AD. The studies showed that AD is lower among patients with DM, odds ratio, 0.69, 95% CI, and 0.67-0.72. No heterogeneity was observed (Chi-Square, 4.12, degree of freedom (df.)= 5, and *I^2 ^*= 0%, P-value), 0.53 and P-value for overall effect, <0.001. The included studies were published in Europe (five) and Canada (one study) and included 162,882 patients and 12,164 events, four of the studied articles were case-control studies, one retrospective, and one cross-sectional.

Conclusion: AD was lower among patients with DM compared to their counterparts without the disease. Further studies focusing on the genetic and environmental factors linking AD and diabetes are needed.

## Introduction

A large upsurge in the prevalence of diabetes mellitus (DM) has been observed worldwide (462 million, 6.3% of the population) and is reflected by increasing rates of diabetes complications and other diabetes-associated diseases. The burden of diabetes and its complications in addition to the accompanied infections and cardiovascular disease is great on the patients and the whole community [1,2]. The pathophysiology of this leading cause of morbidity and mortality is insulin deficiency and/or insulin resistance. DM is on the rise globally. However, and due to the unwanted side effects of pharmacotherapy, non-compliance, and treatment failure, the majority of those suffering from DM are not approaching the recommended targets [3]. Gene therapy and beta-cell regeneration might be potential therapies for DM. Atopic dermatitis (AD) is common and may affect 20% of the general population depending on age and geographical location and AD is not only a disease of childhood as previously thought. It is thought of as a systemic disease and is associated with various diseases including DM [4,5]. The demarcation between type 1 and type 2 diabetes is currently hazy, type 2 diabetes is increasingly observed in the young age group, and more cases of type 1 diabetes are observed among adults [6]. The association between AD and DM is a matter of controversy. Besides, it might be difficult to differentiate between the two most common types of DM at least during the early presentation. Also, an increasing rate of DM is expected during the COVID-19 pandemic due to psychological stressors, barriers to physical activity, and sleep disturbances [7]. Recognizing the associations of AD is vital for patient-centered holistic care. Therefore, the current meta-analysis aimed to assess the relationship between DM and AD.

## Materials and methods

We systematically searched PubMed including Epub and ahead of print (198 articles identified) and Cochrane (13 articles) databases. The searching engine was set to include retrospective and prospective cohorts, case-control studies, and cross-sectional studies. Randomized control trials are not expected due to the nature of the diseases. The studies were approached if they were published in English (difficulty in the translation of other languages) and compare the prevalence of atopic dermatitis among patients with DM and vise versa. We did not specify any type of DM. However, six out of the eight studies included investigated type 1 diabetes, one assessed the rate of type 2 diabetes, and one did not specify DM type.

**Figure 1:**
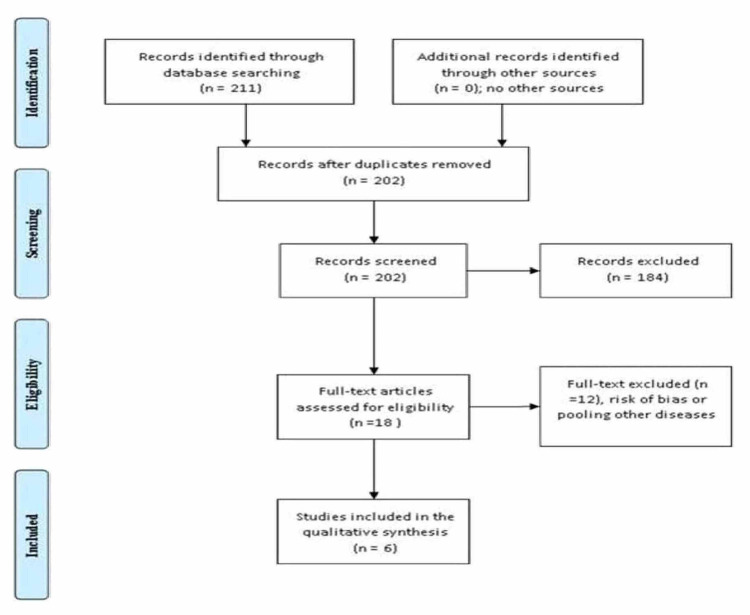
The Association of Diabetes Mellitus and Atopic Dermatitis

Two investigators (H. M. and M. A.) screened the titles and abstracts independently and the references of the six full texts included in the final analysis were screened. Expert's opinions were thought before conducting the review process. The terms used were, AD, DM, type 1 diabetes, type 2 diabetes; discrepancies between the two authors were solved by agreement. No limitations were applied concerning the time of publication of studies (from the first published up to February 13, 2021). In the current meta-analysis, we included prospective, retrospective studies, case-control, and cross-sectional studies conducted on humans and published in the English languages. Studies on animals, experimental studies, and case reports were excluded. Two hundred and eleven were identified, eighteen full texts were screened, six were included in the final meta-analysis. A datasheet was used to record the author's name, year of publication, country and type of the studies, number of events, and total number in the two arms (patients and controls). The primary and secondary outcomes were AD and DM, respectively. The included studies were assessed by the Ottawa Newcastle for cohorts and case-control studies, Ottawa Newcastle Scale is an easy and convenient tool, its validity and reliability have been previously documented. The authors of the included studies were not contacted for more information [8]. Figure 1 depicted the different phases of the systematic review.

Statistical analysis

We used the RevMan system (version 5.4, Cochrane Collaboration, the Nordic Cochrane Centre, Copenhagen) and all the data were dichotomous. Eight studies were included in the preliminary analysis. The random effect was chosen due to the considerable heterogeneity observed. In the current meta-analysis, we investigated the considerable heterogeneity and a sub-analysis was conducted; two studies were removed [9,10]. The former had a long study period of 48 years and assessed various vascular risk factors among wide age groups (3-71 years), while the latter included also asthma and hay fever among twins. The Chi-square and Funnel plot were used to assess heterogeneity. Heterogeneity of <50% was considered significant.

## Results

**Figure 2:**
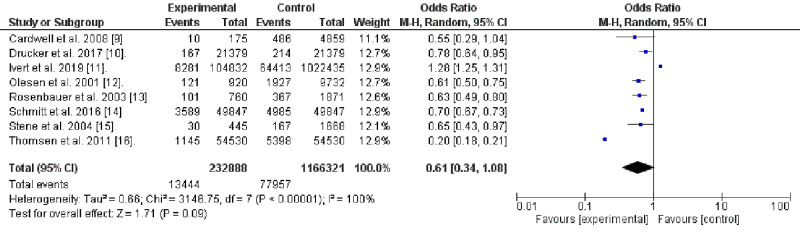
Association of Diabetes Mellitus and Atopic Dermatitis

**Figure 3:**
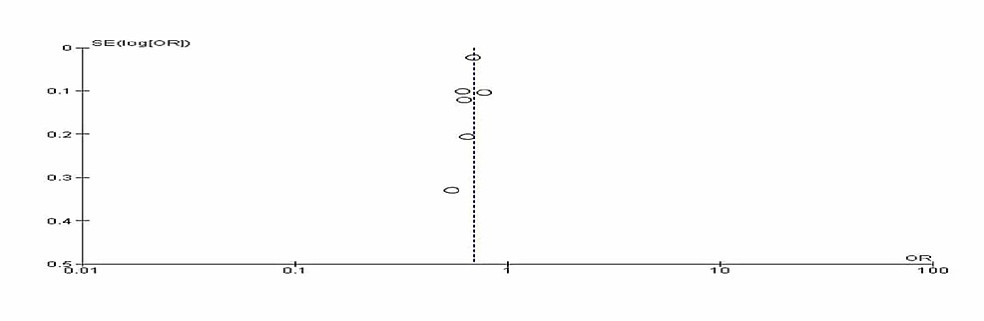
Funnel Plot Comparison: Atopic Dermatitis and Diabetes Mellitus

In the current meta-analysis, out of the 211 articles identified, eight studies were pooled to test the association of DM and AD; two of them were excluded due to the high risk of bias [9,10]. Thus, six surveys were included in the results. All the studies showed that AD is lower among patients with DM [11-16]. The results imply that AD might be protective from DM (odds ratio, 0.69, 95% CI, 0.67-0.72). No heterogeneity was observed (Chi-square, 4.12, degree of freedom (df.)= 5, and I2 = 0%, P-value), 0.53 P-value for overall effect, <0.001. The included studies were published in Europe (five) and Canada (one study) and included 162,882 patients and 12,164 events, four of the studied articles were case-control studies, one retrospective, and one cross-sectional (Table 1, Figures 2 and 3).

**Table 1:** The Interaction of Atopic Dermatitis and Diabetes Mellitus

Author	Country	Methodology	Patients/control	Results
Cardwell et al. [11]	United Kingdom	A case-control	10/175 vs. 486/4859	Risk reduction of type 1 DM
Drucker et al. [12]	Canada	A cross-sectional	167/21379 vs. 214/21379	Risk reduction of type 2 DM
Olesen et al. [13]	Denmark	A case-control	121/920 vs. 1927/9732	A lower rate of type 1 DM
Rosenbauer et al. [14]	Germany	A case-control	101/760 vs. 367/1871	AD was commoner among patients with type 1 DM
Schmitt et al. [15]	Germany	Retrospective	3589/49847 vs. 4985/49847	An inverse relationship between AD and type 1 DM
Stene et al. [16]	Norway	A case-control	30/545 vs. 167/1668	An inverse relationship between AD and type 1 DM

## Discussion

Addressing the associations and comorbidities of AD is essential for multi-disciplinary patient-centered healthcare. The current meta-analysis showed a lower rate of AD among patients with DM. The association of AD with cardiovascular risk factors including DM is conflicting; an interesting finding is the association of the cardiovascular T-helper cell type 1 disease with severe AD [9], another study found a negative association of AD and diabetes genetic factors substantiating the T-helper type-1 vs T-helper type-2 cell dichotomy for these two common morbid disorders [10]. A meta-analysis that investigated the association of AD with cardiovascular risk found no relationship between AD and DM. However, the study assessed unspecified (suspected cases) type 2 diabetes [17]. Addressing the association of AD with cardiovascular risk factors is relevant because of the multi-directional relationship between them. Furthermore, cardiovascular risk factors usually present together in the same patient with deleterious consequences. It is interesting to note that animal studies showed the effectiveness of pioglitazone (an oral hypoglycemic drug) in AD treatment; the proposed mechanisms are restoring the epidermal barrier, reduction of inflammatory cell migration, and pro-inflammatory cytokines modulation [18]. Investigating the association of AD and diabetes is important and paves the way for understanding the shared genetic factors to a better understanding of the pathophysiology. Also, environmental factors that might explain the rapid rise reported in both disorders can be uncovered [19]. The association of chronic autoimmune inflammation with Th-1 deficient diseases like hepatitis-B virus infection and the reverse in Th-1 predominant (type 1 diabetes) has been previously documented, while the role of the previous association with glycemic control and disease progression lacks [20]. Recent epidemiological studies showed an increasing rate of type 1 diabetes with substantial differences according to age, geographical variation, and ethnicities. Importantly, countries with lower incidence showed a steep upsurge of type 1 diabetes, while those with higher rates tend to have a moderate increase or stability [21]. Insulin resistance and the hygiene hypothesis were among the proposed mechanisms that are thought to be behind the increasing rates of type 1 diabetes and great geographical variations observed. The current meta-analysis strength is that it is the first to address the relationship between AD and diabetes raising the concept of the hygiene hypothesis. The interplay between the skin, gut, and microbiota is currently a big area of research. A strong piece of evidence is there about the association of gut microbiota and DM. In fact, fecal transplantation from lean subjects without DM improved insulin sensitivity among patients with metabolic syndrome [21]. We are amid the COVID-19 pandemic with its consequences including antibiotic use, body distancing, travel barrier, and extensive sanitation might alter the gut microbiota [22]. The effects of COVID-19 on reshaping the incidence of AD and DM lack supportive evidence and remained to be answered. The study was limited by including only observational studies, the limitation to the English language, and the unlimited literature search.

## Conclusions

Atopic dermatitis was lower among patients with diabetes pointing to a protective role. Although it is difficult to conclude a cause and effect. Nevertheless, the negative association was documented. Measures to preserve and restore the gut microbiota population for the prevention of various diseases including AD and DM might be appropriate especially during the COVID-19 pandemic and the new families observed.
